# Urinalysis and Urinary Tract Infection: Update for Clinicians

**DOI:** 10.1155/S1064744901000412

**Published:** 2001

**Authors:** Jennifer L. Young, David E. Soper

**Affiliations:** Medical University of South Carolina Gynecology&Obstetrics 96 Jonathan Lucas St. Suite 634 P.O. Box 250619 Charleston SC 29425 USA

## Abstract

Dysuria is a common presenting complaint of women and urinalysis is a valuable tool in the initial evaluation of this presentation. Clinicians need to be aware that pyuria is the best determinate of bacteriuria requiring therapy and that values significant for infection differ depending on the method of analysis. A hemocytometer yields a value of ≥ 10 WBC/ mm^3^ significant for bacteriuria, while manual microscopy studies show ≥ 8 WBC/high-power field reliably predicts a positive urine culture. In cases of uncomplicated symptomatic urinary tract infection, a positive value for nitrites and leukocyte esterase by urine dipstick can be treated without the need for a urine culture. Automated urinalysis used widely in large volume laboratories provides more sensitive detection of leukocytes and bacteria in the urine.With automated microscopy, a value of > 2 WBC/hpf is significant pyuria indicative of inflammation of the urinary tract. In complicated cases such as pregnancy, recurrent infection or renal involvement, further evaluation is necessary including manual microscopy and urine culture with sensitivities.


*Infect Dis Obstet Gynecol 2001;9:249–255*

### Urinalysis and urinary tract infection: update for clinicians

**Jennifer L. Young and David E. Soper**

Medical University of South Carolina, Charleston, SC

Dysuria is a common presenting complaint of women and urinalysis is a valuable tool in the initial evaluation of this presentation. Clinicians need to be aware that pyuria is the best determinate of bacteriuria requiring therapy and that values significant for infection differ depending on the method of analysis. A hemocytometer yields a value of ≥ 10 WBC/mm$^3$ significant for bacteriuria, while manual microscopy studies show ≥ 8 WBC/high-power field reliably predicts a positive urine culture. In cases of uncomplicated symptomatic urinary tract infection, a positive value for nitrites and leukocyte esterase by urine dipstick can be treated without the need for a urine culture. Automated urinalysis used widely in large volume laboratories provides more sensitive detection of leukocytes and bacteria in the urine. With automated microscopy, a value of > 2 WBC/hpf is significant pyuria indicative of inflammation of the urinary tract. In complicated cases such as pregnancy, recurrent infection or renal involvement, further evaluation is necessary including manual microscopy and urine culture with sensitivities.

Key words: Dysuria; Pyuria; Bacteriuria; Urine Culture; Automated Urinalysis

As many as one in four women experience an episode of dysuria each year, making this one of the most common presenting complaints of women seen by clinicians[1]. Dysuria suggests the diagnosis of cystitis but may be present in other conditions such as vaginitis, chlamydial urethritis or pyelonephritis[2]. Urinalysis is valued as a quick and inexpensive screening method for the presence of a lower urinary tract infection (UTI). Often this test is used in conjunction with or in place of a urine culture in the diagnosis of a UTI. However, a recent study found many practicing clinicians use different standards to determine the presence or absence of a UTI, most not updated on current literature[3]. To further complicate matters, the level of automation in urinalysis is increasing and accepted cut-off values differ for these more sensitive methods. This article will review the components of a standard urinalysis, examine the usefulness of each in the diagnosis of UTI, and bring the clinician up to date on automated urinalysis procedures.

### BACTERIURIA IN DIAGNOSIS

While a urine culture is the gold standard in the diagnosis of a UTI, the limitations of this test have led many physicians to use urinalysis as an initial step in the evaluation of dysuria. Bacteriuria is classically defined as a urine culture with greater than 100 000 cfu/ml of a single microorganism[4]. This value distinguishes patients with asymptomatic bacteriuria from those with a contaminated specimen. However, Stamm and colleagues[5] found that this culture count was insufficient to include almost half of patients with symptoms of dysuria and frequency. In a study of 181 women with symptoms of dysuria and frequency, 102 had ≥ $10^5$ cfu/ml but 79 had < $10^5$ cfu/ml and would not have yielded a positive culture by the standard

Correspondence to: David E. Soper, MD, Medical University of South Carolina, Gynecology & Obstetrics, 96 Jonathan Lucas St. Suite 634, P.O. Box 250619, Charleston, SC 29425, USA. Email: soperde@musc.edu criterion. Further study demonstrated that women with dysuria, pyuria and a colony count of $\geq 10^3$ cfu/ml have a lower UTI[1,5]. The additional problem of urine sample contamination clouds the already muddy picture surrounding urine culture. Several studies found 29–32% of urine cultures were contaminated and concluded that the methodology used in collection does not alter this value[6,7]. Because of these difficulties with urine culture, in addition to the fact that it is slow and expensive, physicians have turned to urinalysis to give a quick analysis of elements in the urine. Of these components, leukocytes in the urine demonstrate inflammation and tissue invasion of the urinary tract, distinguishing infection from colonization or contamination[8]. Pyuria is valued as the single best indicator of bacteriuria that will resolve with antimicrobial therapy. If pyuria is absent, antibiotic therapy is not indicated[9].

### PYURIA IN DIAGNOSIS

Pyuria is therefore the most useful analyte for diagnosis of infection and the clinician has a choice between multiple laboratory tests. The gold standard for the definition of pyuria is the leukocyte excretion rate. A leukocyte excretion rate of > 400 000 cells/h correlates well with symptoms of dysuria and frequency as well as the presence of bacteriuria[10]. Research demonstrated that all abacteriuric patients had an excretion rate of < 400 000 cells/h and 144 of 152 bacteriuric patients had a rate of $\geq$ 400 000 cells/h, yielding a sensitivity of 95% and specificity of 100%[11]. Collection of a 24-h urine is not feasible for the clinic setting. For this reason, the chamber count method is used in the literature for accurate determination of pyuria in place of the leukocyte excretion rate. A value of > 10 cell/$mm^3$ of unspun urine is always associated with a leukocyte excretion rate > 400 000 cells/h[12]. For most physicians, these values are only important in determining the accuracy of urinalysis in the detection of pyuria.

### URINALYSIS PART 1: URINE DIPSTICK

A urinalysis with manual dipstick and manual microscopy is commonly ordered on all patients with the presenting symptom of dysuria. However, some hospitals and many clinicians in practice only perform microscopy if there are any abnormalities found with dipstick analysis. A urine dipstick tests for specific gravity and pH, as well as the presence of urobilinogen, ketones, glucose, hemoglobin, leukocyte esterase and nitrite. The determination of a positive result is based on comparison of color development with the standard strip provided by the manufacturer. The urine dipstick is valued as a quick and inexpensive test that requires little expertise to perform correctly. The results can prove useful in determining whether to treat empirically or to order a urine culture for definitive determination of bacteriuria. The most useful analytes for UTI diagnosis are leukocyte esterase and nitrite.

Leukocyte esterase is an enzyme found in neutrophil granules that reacts with agents on the dipstick to produce a blue color in 1–2 min. Specific positive values vary from trace to many, correlating with a minimum number of WBC/hpf. For the Chemstrip (Boehringer Mannheim, IN) L/N this minimum threshold is > 5 WBC/hpf[13], but this value may vary depending on the test strip. While trace or small leukocyte esterase activity may be considered a positive result for this analyte, this result is not very specific for a UTI as many other conditions can cause pyuria. White blood cells can be found in the urine in a variety of conditions ranging from chlamydial urethritis to pyelonephritis. For this reason, the positive predictive value of leukocyte esterase has been shown to vary between 19 and 88%[14,15]. The absence of leukocyte esterase activity has a negative predictive value of 97–99% indicating that the urine culture will be < $10^3$ cfu/ml[14]. This discrepancy is based on the fact that if the urinary tract is infected, the significant inflammation of the mucosa should produce pyuria. In the absence of pyuria, the diagnosis of a UTI is unlikely.

Nitrite is produced from dietary nitrate by bacteria containing nitrate reductase. The amine in the dipstick reacts with the nitrite and produces a blue color in 60 s. The presence of nitrites is highly specific for bacteriuria (96.6–97.5%), but has a low sensitivity of 0–44% for bacteriuria between $10^3$ and $10^5$ cfu/ml[13,14]. False negatives may be the result of a lack of dietary nitrate, dilution of the nitrite in the urine (such as with diuretics), or non-nitrate-reducing bacteria including *Staphylococcus*, *Enterococcus*, or *Pseudomonas*[16]. VanNostrand and colleagues[17] found that 78.9% of samples containing nitrate-reducing bacteria were actually negative for nitrite on the urine dipstick. Therefore the presence of nitrite has a high positive predictive value of 94%[18], but in the absence of urinary nitrite a UTI cannot be ruled out.

Taking the leukocyte esterase and nitrite together proves much more useful. If both tests are positive, the specificity increases to 98–99.5%, indicating a high likelihood of a UTI[14,19]. Several studies conclude that from this information alone a diagnosis of a UTI can be made and treatment initiated[2,18,20,21]. This clinical conclusion has recently been called into question by a study demonstrating a low sensitivity for both leukocyte esterase and nitrite but did not take into account the clinical presentation of the patients in the study[17]. In a low prevalence population, such as in screening asymptomatic patients, the sensitivity of these tests can be as low as 56%[22]. From this data, Lachs and colleagues[22] concluded that in all future studies concerning the diagnosis of UTI, clinical information including symptomatology must be included. Therefore, if both analytes are positive and the patient is a symptomatic female, the prevalence in this population is sufficiently high that the positive predictive value is drastically increased. If both analytes are negative, the chances of infection fall to 0–5%[14,23]. Of note, the colors of the urine dipstick will spontaneously change if allowed to develop in air for greater than 15 min[13]. Urine dipstick must be performed in a timely manner to ensure accurate results.

### URINALYSIS PART 2: MICROSCOPY

Microscopy is a valuable addition to the urine dipstick in diagnosing a UTI and should be performed when using urinalysis to make a treatment decision. Manual microscopy is still widely used to determine pyuria despite knowledge that the results are highly variable depending on the laboratory, technician and urine sample[24]. When preparing a slide, the urine specimen is centrifuged, the supernatant decanted, and the sediment is resuspended. This is typically the least standardized portion of the procedure with several studies suggesting centrifuging 10–20 ml urine at varying speeds for approximately 5 min, then resuspending the sediment in a drop up to 0.2 ml supernatant[16,24,25]. The technician places the sediment on the slide and views between 5 and 15 fields before reporting an average value of WBC/hpf. Visualization of the formed elements in the urine is the greatest benefit of microscopy. Microscopic evaluation of leukocytes has a positive predictive value of 100% for the presence of pyuria (defined as $> 8$ WBC/mm$^3$) but does not correlate as clearly with the presence of bacteriuria. However, as the number of cells/hpf increases, the positive predictive value for bacteriuria increases[26]. Microscopy can also be used to visualize bacteria in the urine. Using a counting chamber, Hiraoka and colleagues[27] found that the presence of both bacteriuria and pyuria had a positive predictive value of 100% compared with the urine culture while the absence of both had a negative predictive value of 100%. Microscopy is also valued for visualization of casts or crystals that may indicate renal involvement or a complicated UTI.

However, there are many drawbacks to this useful test. Studies show that cells are lost in the handling of the sample and the transfer onto the slide[28,29]. Manual microscopy is the least standardized and most time-consuming portion of urinalysis[30]. Because of this, many hospitals have resorted to examining the urine under microscope only if the urine dipstick is negative. Unfortunately, this negates the value of microscopy as a tool both to increase the sensitivity of urinalysis and to directly visualize the elements in the urine. The solution to this dilemma may be in new techniques for automation of microscopy and urine dipstick that allow more standardization and decrease the number of samples that must be reviewed by manual microscopy.

### AUTOMATED URINALYSIS

Automated instruments for urinalysis have entered hospital and reference laboratories and may soon find their way to clinics. Due to discrepancies among technicians, laboratories and hospitals, automated techniques have been introduced in the past 25 years to update urinalysis to the level of other standardized laboratory procedures. Automation exists on several levels. In a survey of local hospitals, we found that most hospitals employ an automated method for dipstick analysis but still perform a manual microscopic evaluation. The instrument typically reads the chemistries contained on the dipstick by spectroscopy, reporting these values to a computer interface. At the highest level of automation, the instrument performs the dipstick analysis as well as a count of the formed elements in the urine. It takes up unspun, stained urine in a thin layer and analyzes it by flow microscopy. Stop motion pictures are taken of each field and the computer processes the images, sorts particles based on size, and counts them. The operator then identifies the particles present and the instrument reports the number of cells per high power field.

Comparisons of both automated dipstick and automated microscopy with manual methods show good correlation between the two. Automated analysis of the urine dipstick has been shown to have comparable results with a manual dipstick except for specific gravity, which was better measured manually[31]. Automated microscopy also correlates well with manual microscopy for most urine elements with the exception of casts. Significantly more urine casts were found using manual microscopic evaluation[29,31,32]. One report indicated a sensitivity of 26–69% in automated detection of casts and recommended use of manual microscopy when renal damage is suspected[33]. Studies have shown that automated instruments are more sensitive in the detection of both red blood cells and white blood cells[34–36]. Leukocyte counts correlated well with manual microscopy in most studies and also predicted bacteriuria at a value of > 2 cells/hpf[36]. The automated urinalysis system is especially useful at a lower range of cell counts where the dipstick is insensitive and the manual microscopic count is imprecise[32]. The additional sensitivity may be due in part to the fact that the specimen is not centrifuged before analysis. One study found an additional 50% of abnormal urine elements with automated urinalysis over manual microscopy[34]. However, a positive result in a low incidence population such as asymptomatic patients would require further testing. In addition to increased sensitivity, the laboratory and hospital benefit from the technology in a decrease in turn-around time, labor and cost.

### COST ANALYSIS

In discussing the usefulness of urinalysis, one must discuss the issue of cost. UTIs prompt over 5.2 million doctors' office visits per year yielding a total US health-care cost of over 1 billion dollars[2]. Cutting the costs in the diagnosis of a UTI can result in significant savings in medical care. As previously mentioned, urinalysis is valued because it is an inexpensive alternative to ordering a urine culture on every patient with urinary complaints. Currently, a urine culture costs $35 while simple urinalysis costs as little as $4. Automated urinalysis at our institution costs the patient $13. Using urinalysis to guide which urine samples to culture can mean significant savings. Automated urinalysis has also been proposed to cut down on the cost associated with the diagnosis of a UTI. While the initial expense of the equipment must be considered, this is balanced against a drastic decrease in labor cost. Automation reduces the turn-around time for each urinalysis, predominantly by decreasing the number of urine samples that must be viewed under manual microscopy[32]. Bartlett and colleagues[36] performed a cost analysis comparing the Yellow IRIS (IRIS, Chatsworth, CA) as a screening test to obtaining a urine culture on all samples submitted[36]. The investigators assumed a $16.32/h labor cost and found that to screen each urine sample would cost 24¢, using 0.3 min for accession, 0.5 min for analysis, and 0.1 min for reporting results. This study demonstrated that approximately 50% of the samples would require a urine culture and still found significant savings by screening with automated urinalysis[36]. In determining the savings to our institution and our patients, we used a urine culture cost and urinalysis cost given above. It would cost a laboratory receiving 25 000 urine samples/year $875 000 to screen via urine culture and only $325 000 to perform a urinalysis on each sample. If the urinalysis is positive for both significant leukocytes and bacteria in the urine, no further testing is warranted and the urinalysis results can be considered diagnostic for a UTI. Should the urinalysis report bacteriuria without pyuria or the reverse then a urine culture should be obtained to rule out a UTI. Even if 50% of these samples require further characterization with a urine culture (based on the results of the urinalysis and the prevalence of UTI in that patient population) the total savings would exceed \$110 000 per year. This figure justifies the expense of automated urinalysis workstations and advocates the use of these machines not only to reduce cost in diagnosing a UTI but also to enhance the reliability of urinalysis as both a screening and diagnostic tool.

### USING URINALYSIS TO DIAGNOSE A UTI

Most physicians order a urinalysis for any patient with the presenting symptom of dysuria and may only order a urine culture on those with a positive urinalysis. The history may suggest a particular diagnosis and this pretest probability must be taken into account in evaluation of the urinalysis results (Table 1[37]). It has been suggested in the literature that a positive urinalysis for both nitrites and leukocyte esterase activity for a symptomatic patient does not require further testing. A urine culture is not warranted in these cases for several reasons: the finding of pyuria and bacteriuria is 99.5% specific for a UTI, most urinary pathogens are susceptible to common antimicrobial therapy, and the possibility of increased morbidity (associated with lower sensitivity) is low for uncomplicated cases[20]. Manual microscopy should not be overlooked, even in the setting of automated urinalysis, if renal damage is suspected. In this instance, visualization of the urine would be necessary to look for specific casts or crystals that may point to particular renal pathology.

There are specific circumstances in which urinalysis is insufficient and urine culture must be used in making treatment decisions. Physicians should order a urine culture on all patients with symptoms suggestive of pyelonephritis or symptoms persisting 3 days after the initiation of therapy. Further, a urine culture should be performed in all cases of recurrent infection to determine the organism and its antibiotic sensitivities[1]. It may be that the original infection involved an unusual or resistant organism. In a low prevalence population, such as in screening for asymptomatic bacteriuria of pregnancy, a urinalysis is not sensitive enough to rule out infection and a urine culture is necessary to determine the presence or absence of a UTI[38–40]. In this population only 4–7% of patients may actually have a UTI but the associated significant morbidity to both mother and fetus warrants the expense of screening each patient with a urine culture[39].

Whether manual or automated, urinalysis will help determination of the presence or absence of leukocytes and bacteriuria in the urine. With this knowledge, combined with the clinical presentation of the patient and the pretest probability of a UTI, the clinician can decide whether to order a urine culture or treat on the basis of these data. Pyuria is the best indicator of those patients with bacteriuria corresponding with active infection of the urinary tract. Practicing clinicians need to be aware of the method of urinalysis being performed and the values of pyuria that correlate with infection (Table 2).

**Table 1** Signs and symptoms of urinary tract infection. Comparison of symptoms to a positive urine culture[37]

| | *Positive predictive value* | *Specificity* |
|---|---|---|
| Frequency | 58% | 6% |
| Lower abdominal pain | 59% | 36% |
| Loin pain | 60% | 68% |
| Dysuria | 65% | 28% |
| Hematuria | 72% | 87% |
| Fever | 78% | 85% |

**Table 2** Correlation of pyuria with bacteriuria

| | *Cut-off value* | *Bacteriuria (cfu/ml)* |
|---|---|---|
| Leukocyte excretion rate[10] | 400 000 cells/h | 95% ($\geq 10^4$) |
| Hemocytometer[8] | $\geq$ 10 cells/mm$^3$ | > 96% ($\geq 10^5$) |
| Leukocyte esterase activity[41] | small | 84% ($\geq 10^3$) |
| Manual microscopy[26] | $\geq$ 8 cells/hpf | 82% ($\geq 10^3$) |
| Automated microscopy[36] | $\geq$ 2 cells/hpf | 93% ($\geq 10^4$) |

### REFERENCES

1. Komaroff AL. Acute dysuria in women. *N Engl J Med* 1984;310:368–74
2. Powers RD. New directions in the diagnosis and therapy of urinary tract infections. *Am J Obstet Gynecol* 1991;164:1387–9
3. Wigton RS, Longnecker JC, Bryan TJ, *et al.* Variation by specialty in the treatment of urinary tract infection in women. *J Gen Intern Med* 1999;14: 491–4
4. Brumfitt W. Urinary cell counts and their value. *J Clin Pathol* 1965;18:550–5
5. Stamm WE, Wagner KF, Amsel R, *et al.* Causes of the acute urethral syndrome in women. *N Engl J Med* 1980;303:409–15
6. Lifshitz E, Kramer L. Outpatient urine culture: does collection really matter? *Arch Intern Med* 2000; 160:2537–40
7. Valstein P, Meier F. Urine culture contamination: a College of American Pathologists Q-Probes study of contaminated urine cultures in 906 institutions. *Arch Pathol Lab Med* 1998;122:123–9
8. Stamm WE. Measurement of pyuria and its relation to bacteruria. *Am J Med* 1983;75(1B):53–8
9. Stamm WE, Running K, McKevitt M, *et al.* Treatment of the acute urethral syndrome. *N Engl J Med* 1981;304:956–8
10. Mabeck. Studies in urinary tract infections. *Acta Med Scand* 1969;186:193–8
11. Little PJ. Urinary white cell excretion. *Lancet* 1962;1:1149
12. Little PJ. A comparison of the urinary white cell concentration with the white cell excretion rate. *Br J Urol* 1964;36:360–3
13. Oneson R, Groschel DH. Leukocyte esterase activity and nitrite test as a rapid screen for significant bacteriuria. *Am J Clin Pathol* 1985;83:84–7
14. Semeniuk H, Church D. Evaluation of the leukocyte esterase and nitrite urine dipstick screening tests for detection of bacteriuria in women with suspected uncomplicated urinary tract infections. *J Clin Microbiol* 1999;37:3051–2
15. Barlett RC, Zern DA, Ratiewicz I, Tetreault JZ. Reagent strip screening for sediment abnormalities identified by automated microscopy in urine from patients suspected to have urinary tract disease. *Arch Pathol Lab Med* 1994;118:1096–101
16. Pappas PG. Laboratory in the diagnosis and management of urinary tract infections. *Med Clin North Am* 1991;75:313–25
17. VanNostrand JD, Junkins AD, Bartholdi RK. Poor predicitive ability of urinalysis and microscopic examination to detect urinary tract infection. *Am J Clin Pathol* 2000;113:709–13
18. Blum RN, Wright RA. Detection of pyuria and bacteriuria in symptomatic ambulatory women. *J Gen Intern Med* 1992;7:140–4
19. Hussain R, Chaudry NA, Anwar MS, *et al.* Evaluation of dipstrips, direct Gram stain and pyuria as screening tests for the detection of bacteriuria. *J Pakist Med Assoc* 1996;46:38–41
20. Komaroff AL. Urinalysis and urine culture in women with dysuria. *Ann Intern Med* 1986;104: 212–18
21. Jellheden B, Norrby RS, Sandberg T. Symptomatic urinary tract infection in women in primary health care. Bacteriological, clinical, and diagnostic aspects in relation to host response to infection. *Scand J Prim Health Care* 1996;14:122–8
22. Lachs MS, Nachamkin I, Edelstein PH, *et al.* Spectrum bias in the evaluation of diagnostic tests: lessons from the rapid dipstick test for urinary tract infection. *Ann Intern Med* 1992;117:135–40
23. Holland DJ, Blis KJ, Allen CD, Gilbert GL. A comparison of chemical dipsticks read visually or by photometry in the routine screening of urine specimens in the clinical microbiology laboratory. *Pathology* 1995;27:91–6
24. Winkel P, Statland BE, Jorgensen K. Urine microscopy, an ill-defined method, examined by a multifactorial technique. *Clin Chem* 1974;20:436–9
25. Lorincz A, Kelly DR, Dobbins GC, *et al.* Urinalysis: current status and prospects for the future. *Ann Clin Lab Sci* 1999;29:169–75
26. Holm S, Andres W, Wahlqvist L, *et al.* Urine microscopy as a screening method of bacteriuria. *Acta Med Scand* 1982;211:209–12
27. Hiraoka M, Hida Y, Hori C, *et al.* Urine microscopy on a counting chamber for diagnosis of urinary tract infection. *Acta Paediatr Jpn* 1995;37: 27–30
28. Gadeholt H. Quantitative estimation of cells in the urine. *Acta Med Scand* 1968;183:369–74
29. Langlois MR, Delanghe JR, Steyaert SR, *et al.* Automated flow cytometry compared with an automated dipstick reader for urinalysis. *Clin Chem* 1999;45:118–22
30. Lakatos J, Bodor T, Zidarics Z, Nagy J. Data processing of digital recordings of microscopic examination of urinary sediment. *Clin Chim Acta* 2000; 297:225–37
31. Elin R, Housseini JM, Kestner J, *et al.* Comparison of automated and manual methods of urinalysis. *Am J Clin Pathol* 1986;86:731–7

32. Fenili D, Pirovano B. The automation of sediment urinalysis using a new urine flow cytometer (UF-100). *Chem Lab Med* 1998;36:909–17
33. Kouri T, Kahkonen U, Malminiemi K, *et al.* Evaluation of Sysmex UF-100 urine flow cytometer vs chamber counting of supravitally stained specimens and conventional bacterial cultures. *Am J Clin Pathol* 1999;112:25–35
34. Carlson DA, Statland BE. Automated urinalysis. *Clin Lab Med* 1988;8:449–61
35. Ben-Ezra J, Bork L, McPherson RA. Evaluation of the Sysmex UF-100 automated urinalysis analyzer. *Clin Chem* 1998;44:92–5
36. Bartlett R, Zern DA, Ratkiewicz I, Tetreault JZ. Screening for urinary tract infection with the Yellow IRIS. *Lab Med* 1992;23:599–602
37. Sanford JP. Urinary tract symptoms and infections. *Annu Rev Med* 1975;26:485–98
38. Tincello DG, Richmond DH. Evaluation of reagent strips in detection of asymptomatic bacteriuria in early pregnancy: prospective case series. *Br Med J* 1998;316:435–7
39. Patterson TF, Andriole VT. Detection, significance, and therapy of bacteriuria in pregnancy. *Infect Dis Clin North Am* 1997;11:593–608
40. Bachman JW, Heise RH, Naessens JM, Timmerman MG. A study of various tests to detect asymptomatic urinary tract infections in an obstetric population. *J Am Med Assoc* 1993;270:1971–4
41. Pfaller M, Ringenberg B, Rames L, *et al.* The usefulness of screening tests for pyuria in combination with culture in the diagnosis of urinary tract infection. *Diagn Microbiol Infect Dis* 1987;6:207–15

RECEIVED 02/05/01; ACCEPTED 06/22/01
